# Evaluation of the Short-Term Effects of Mirtazapine on Appetite Stimulants in Dogs: A Retrospective Study and a Placebo-Controlled Trial

**DOI:** 10.3390/ani15172538

**Published:** 2025-08-29

**Authors:** Stephanie S. Theodoro, Maria Eduarda G. Tozato, Thais O. Ximenes, Lara M. Volpe, Camila Baptista da Silva, Fabio A. Teixeira, Aulus C. Carciofi

**Affiliations:** 1 Veterinary Medicine and Surgery Department, College of Agrarian and Veterinarian Sciences (FCAV), São Paulo State University–UNESP, Jaboticabal 14884-900, SP, Brazil; m.tozato@unesp.br (M.E.G.T.); thais.ximenes@unesp.br (T.O.X.); laramv.vet@gmail.com (L.M.V.); camila_bds@hotmail.com (C.B.d.S.); aulus.carciofi@unesp.br (A.C.C.); 2 Graduate Program in Animal Production, University of Brasil, Fernandópolis 15600-000, SP, Brazil; 3 School of Veterinary Medicine and Animal Science, University of São Paulo-Brazil, São Paulo 05508-270, SP, Brazil; fabioa14@hotmail.com

**Keywords:** anorexia, hyporexia, orexigenic, nutrition

## Abstract

Anorexia and hyporexia are common clinical signs in dogs with acute or chronic illnesses, often requiring targeted nutritional interventions. Mirtazapine, an orexigenic drug widely used in cats, has limited evidence supporting its use in dogs. This study evaluated mirtazapine’s efficacy as an appetite stimulant in dogs using a two-part approach: (1) a retrospective analysis of 107 clinical cases comparing dogs that received mirtazapine to untreated controls; and (2) a prospective, placebo-controlled, double-blind crossover trial involving 25 dogs. In the retrospective analysis, dogs treated with mirtazapine were significantly more likely to resume voluntary food intake (OR = 3.06) and had less weight loss compared to controls. In the prospective trial, mirtazapine significantly increased food acceptance on the first day (100% vs. 63.6%, *p* = 0.03), with no adverse effects observed. However, the effect was not maintained on the second day, possibly due to residual drug activity or clinical improvement. Latency to feeding averaged 120 min post-administration. Mirtazapine was safe and well tolerated, even in dogs with comorbidities. These findings support the short-term use of mirtazapine as a second-line appetite stimulant in dogs when enteral feeding is not feasible.

## Simple Summary

Many sick dogs lose their appetite, which makes it harder for them to recover. Feeding tubes are often the best solution, but not all pet owners agree to use them. Mirtazapine is a medicine already used in cats to help stimulate appetite, but there is limited information about its use in dogs. This study evaluated how well mirtazapine works in dogs with reduced appetites. First, the medical records of over 100 dogs were reviewed, which showed that dogs who received mirtazapine were more likely to start eating again. Then, in a controlled study with 25 dogs, those who received mirtazapine ate more food than those who received a placebo on the first day. The medicine was safe and did not cause side effects. These results suggest that mirtazapine can be a helpful and safe option to encourage eating in sick dogs when other methods are not possible, especially in the short term.

## 1. Introduction

The prevention and reversal of malnutrition are key goals of therapeutic intervention, as maintaining adequate nutritional status is essential for clinical recovery and patient survival. Inappetence (including hyporexia and anorexia) is a common clinical sign in hospitalized dogs and cats or those suffering from chronic diseases and it is strongly associated with deterioration of nutritional status and a poorer prognosis [1,2,3].

The behavioral expression of appetite is complex and can be modified by altered sense of taste and smell, dysphagia, pain and learned food aversions, as well as alteration in hormone and cytokine levels within the body [4]. Various clinical conditions, including inflammatory diseases, metabolic disorders, and trauma, can lead to reduced food intake, requiring specific nutritional strategies to mitigate the risk of catabolism and loss of body mass [2,4,5].

Recommended approaches to stimulate voluntary food consumption include the provision of highly palatable diets with increased moisture, fat, and protein content [6,7], as well as effective control of pain and associated symptoms such as nausea and vomiting [2,8]. When these measures are insufficient, early implementation of nutritional support via enteral feeding tubes becomes essential to prevent a negative energy balance [9].

In clinical practice, feeding tubes are often underutilized due to hesitation or lack of familiarity among veterinarians, but primarily because of the owners’ refusal to authorize their use. Therefore, alternative techniques are necessary to ensure adequate nutritional support. Force-feeding is never indicated, as it may increase the risk of aspiration, lead to food aversion, and fail to ensure adequate caloric intake [1,2,9].

Several medications have been employed as option to stimulate appetite. Although anabolic steroids, corticosteroids, and benzodiazepines had been historically used as appetite stimulants, their use has been discontinued due to significant adverse effects [10]. Other medications such as cyproheptadine [7], capromorelin [11], and mirtazapine [12] are used to stimulate appetite.

Among these orexigenic agents employed in small animal clinical practice, mirtazapine is particularly noteworthy due to antiemetic and appetite stimulant especially in cats with decreased appetite and weight loss associated mainly with chronic kidney disease [12,13]. These properties are mediated by inhibition of serotonergic 5-HT3, 5-HT2A, and 5-HT2C receptor [14,15]. Mirtazapine is a presynaptic α2-adrenergic antagonist, which increases the release of norepinephrine and serotonin, contributing to appetite stimulation [16,17].

Moreover, the appetite-stimulating effects of mirtazapine have also been demonstrated in other species, further supporting its clinical relevance. In cats, several studies have confirmed its efficacy both in oral and transdermal formulations, particularly in patients with chronic kidney disease, where it not only increased food intake but also promoted weight gain [12,18,19]. Beyond felines, positive outcomes have been reported in rabbits, where oral administration was shown to be safe and effective in enhancing appetite [20], and in humans, where mirtazapine has been associated with weight gain as a secondary effect of long-term treatment for depression [21]. Collectively, these findings provide strong evidence across different species that mirtazapine consistently exerts orexigenic properties, reinforcing its potential role as an appetite stimulant in dogs.

Despite advances in the clinical use of mirtazapine, there remains a lack of systematic data evaluating its efficacy and safety across different clinical scenarios in veterinary medicine, particularly in dogs. Thus, the present study aimed to assess the orexigenic effects of mirtazapine in dogs in general clinical practice.

## 2. Material and Methods

### 2.1. Study 1–Retrospective Evaluation

This retrospective study was based on the review of medical records from Small Animal Clinical Nutrition Service of the “Governador Laudo Natel” Veterinary Hospital at the School of Agricultural and Veterinary Sciences, São Paulo State University, Jaboticabal campus, SP, Brazil (FCAV/UNESP Jaboticabal), between March 2016 and February 2017.

Information was extracted from medical records of dogs presenting anorexia (complete absence of food intake for at least 24 h) or hyporexia (intake of less than 50% of the usual amount for at least 48 h), regardless of the underlying disease. At our Small Animal Clinical Nutrition Service, the standard protocol for evaluating patients whose owners reported inappetence consisted of offering a standardized tray containing dry commercial diets with added palatability enhancers such as heavy cream, as well as a variety of wet diets (including pâtés and chunks in gravy). If the patient consumed ≥50% of the offered amount, voluntary feeding with dietary adjustment was continued. If intake was <50% or absent, the initial recommendation was the placement of a feeding tube for enteral nutritional support. Mirtazapine was considered a second-line option, prescribed when the owner declined tube placement.

To better assess dietary intake, the amount of food to be consumed was also prescribed. Energy requirements were calculated according to the Nutritional Requirements of Dogs for inactive adult dogs: 95 kcal/kg^0.75^ [22,23].

To analyze the effects of mirtazapine use and food consumption, the animals were divided into four groups:

(A) Animals that received mirtazapine and showed a positive effect on increased appetite;

(B) Animals that received mirtazapine and did not have a positive effect on increased appetite—a negative effect;

(C) Animals for which mirtazapine was recommended but not administered due to owner refusal, but showed a positive effect on increased appetite—positive control;

(D) Animals for which mirtazapine was recommended but not administered due to owner refusal, but showed a negative effect on increased appetite—negative control.

Therefore, groups A and B received the orexigenic mirtazapine, and groups C and D did not receive any type of orexigenic.

In all groups, increased food appetite, animal age and weight, body condition score (BCS [24]), muscle mass score (MMS [25]), weight variation, and prescribed mirtazapine dose were assessed.

Data were analyzed using R software (version 4.3.1). Non-parametric tests were employed after confirming non-normal distribution of variables using the Shapiro–Wilk test. Categorical group comparisons (appetite stimulation and weight maintenance) were performed using Fisher’s exact test, with effect size estimated through odds ratios (OR) and 95% confidence intervals (95% CI). Continuous variables (age, body weight, BCS, MMS, and mirtazapine dosage) were analyzed using the Kruskal–Wallis test followed by Dunn’s post hoc test for pairwise comparisons. Weekly weight variation (%) was assessed using one-way ANOVA with Tukey’s post hoc test. A significance threshold of *p* < 0.05 was adopted for all analyses.

### 2.2. Study 2–Prospective Placebo-Controlled Clinical Trial

This study was conducted at the “Governador Laudo Natel” Veterinary Hospital (FCAV/UNESP Jaboticabal). All experimental procedures were approved by the Animal Use Ethics Committee, protocol no. 793/24, and followed the guidelines of the Brazilian College of Animal Experimentation. All owners were informed of the study objectives and provided informed consent.

Dogs evaluated by the Small Animal Clinical Nutrition Service who presented with anorexia or hyporexia lasting from one to three days, regardless of the underlying disease, were included in the study. Only animals able to consume high-protein, high-fat diets—without gastrointestinal or metabolic contraindications—were eligible.

The experimental design consisted of a double-blind, randomized, two-day crossover clinical trial. Randomization was performed in an alternating sequence: the first patient received capsule A on Day 1 and capsule B on Day 2; the second patient began with capsule B on Day 1 and received capsule A on Day 2; and so forth. Each animal served as its own control, which reduced interindividual variability and enhanced the statistical robustness of the analysis. Both treatments were initiated within the Veterinary Hospital, with the Day 1 capsule always administered directly by a veterinarian.

The experimental treatments consisted of mirtazapine capsules and matching placebo capsules; the latter composed of cornstarch. Mirtazapine doses were adjusted on a mg/kg basis to account for individual body weight [16]:

- <7 kg: 3.75 mg every 24 h
- 8–15 kg: 7.5 mg every 24 h
- 16–30 kg: 15 mg every 24 h
- 30 kg: up to 30 mg every 24 h

Throughout the study, all patients received the same highly palatable commercial extruded diet indicated to puppies, formulated with 4200 kcal/kg of metabolizable energy, 76.2 g/1000 kg of crude protein, 47.6 g/1000 kcal of fat, and 5.95 g/1000 kcal of crude fiber.

Monitoring lasted 48 h, with three main assessments:

- Diet consumption after capsule administration (yes or no);
- Percentage of the recommended daily diet not consumed, based on each animal’s maintenance energy requirement (REM = 95 kcal/kg^0.75^);
- Latency time in minutes between capsule administration (mirtazapine or placebo) and the onset of voluntary food intake.

All owners received a standardized monitoring form to be completed over the two-day observation period. This form included specific questions regarding the time of capsule administration, whether the animal accepted food, the exact time of food intake if accepted, whether the prescribed food was consumed or another type of food was offered, and whether the full portion of the prescribed food was consumed. If not, owners were instructed to indicate the amount of food refused. The prescribed food portions were calculated individually and provided in pre-weighed daily amounts. In cases of leftovers, owners were asked to return the remaining food to the hospital so it could be weighed. Alternatively, caregivers who had access to a scale were allowed to weigh the leftovers themselves and report the value on the monitoring form. Although the food was not offered ad libitum, owners were instructed to offer it initially 40 min after capsule administration and then at different times throughout the day in case of initial refusal. They were discouraged from offering other types of food, although some did so when the animal showed no interest in the prescribed diet. Additionally, it was recommended that caregivers maintain a 24-h interval between the administration of the first and second capsules, regardless of treatment. After completing the two-day protocol, all participants returned to the hospital on the third day for a follow-up evaluation and to return the completed form.

All animals continued receiving the conventional treatment prescribed by Small Animal Internal Medicine Service for the underlying conditions causing inappetence.

The data were analyzed using Fisher’s exact tests and chi-square tests to compare the proportions of food leftovers (categorized into 0–25%, 26–50%, 51–75%, and 76–100%) between the mirtazapine and placebo groups on each experimental day. For behavioral variables, we applied Fisher’s exact tests (food acceptance) and paired Wilcoxon tests (latency time). The analyses included calculating odds ratios (OR) with 95% confidence intervals and assessing standardized residuals to identify specific differences. All tests were performed in R (version 4.3.1), with *p* < 0.05 considered statistically significant.

## 3. Results

### 3.1. Study 1

In a one-year retrospective study of clinical appointments at the Small Animal Clinical Nutrition Service of the Teaching Veterinary Hospital, 3063 patients were evaluated across both initial consultations and follow-up visits, comprising 2544 dogs (83.3%). Among these, 566 dogs (21.4%) presented with inappetence lasting ≥24 h secondary to underlying conditions. Enteral feeding tube support was initiated or maintained during appointments for 455 dogs (80.4% of inappetent cases), including both nutritional management (e.g., mandibulectomy recovery) and primary appetite-related indications. Tube types included nasogastric (222 cases, 48.8%), nasoesophageal (35 cases, 7.7%), and esophageal (126 cases, 27.7%). Mirtazapine was prescribed during appointments to 189 inappetent dogs (33.4%); after excluding 82 cases with incomplete follow-up records or missed appointments, the analyzable cohort included 107 dogs—51 receiving mirtazapine and 56 untreated controls (Table 1).

The study population presented with the following primary diagnoses: multisystemic disorders (34.6%, *n* = 37), chronic kidney disease (13.1%, *n* = 14), hemolymphatic disorders (6.5%, *n* = 7), infectious diseases (3.7%, *n* = 4), hemoparasitoses (5.6%, *n* = 6), endocrine disorders (3.7%, *n* = 4), and neoplasms (6.5%, *n* = 7). Demographic characteristics revealed a mean age of 9.02 ± 4.24 years (range: 0.25–17 years), with 52.3% females (*n* = 56) and 47.7% males (*n* = 51), and a mean body weight of 20.25 ± 13.45 kg. No difference was observed between the groups for these variables (Table 2).

Notably, among control group, 37.5% (*n* = 21/56) resumed spontaneous feeding within 72 h, while 10.7% (*n* = 6/56) required enteral feeding support. In animals that received Mirtazapine, the dosage followed the hospital’s clinical protocol, with administered doses ranging from 0.45 to 0.74 mg/kg q24h (0.6 mg/kg). Among dogs that responded positively to treatment, mirtazapine was prescribed for an average of 11.7 days (range: 5–20 days), whereas in non-responders, treatment was maintained for an average of 11.9 days (range: 10–30 days).

Therapeutic outcomes demonstrated significant intergroup differences (Table 2). The mirtazapine group showed superior appetite stimulation (68.6% responders, *n* = 35) compared to controls (37.5%, *n* = 21; *p* = 0.002), with an odds ratio of 3.06 (95% CI: 1.45–6.47). Weight change analysis revealed that mirtazapine responders gained more body weight weekly than non-responders, while control groups showed similar divergence: the positive effect group gained more body weight than negative effect. Although no difference was observed between the responders in the two groups, the animals that lost weight in the control group lost more per week than those in the mirtazapine group. There was no difference in weight, BCS, MMS and the number of animals that maintained their body weight.

### 3.2. Study 2

Thirty animals were initially included in the study. However, five were excluded due to failure to return for follow-up or non-compliance with the provided instructions, resulting in a total of 25 patients who completed the experimental protocol. Fourteen dogs received sequence mirtazapine–placebo, while 11 received placebo–mirtazapine.

This prospective study included animals of twelve breeds: mixed breeds were most common (11/25, 44%), followed by Yorkshire Terriers (3/25, 12%) and Shih Tzus (2/25, 8%). Single cases represented German Shepherd, Border Collie, Beagle, Chihuahua, Pit Bull, Golden Retriever, Poodle, Lhasa Apso, and Pug. The mean (±standard deviation) BCS was 5.04 ± 1.70, and the MMS was 2.04 ± 0.84. The underlying diseases were 32% renal disorders (*n* = 8/25), 20% hepatic conditions (*n* = 5/25) and 20% neoplasms (*n* = 5/25).

The body weight distribution, used to determine the daily mirtazapine dose, was six animals weighed less than 7 kg (mirtazapine dosage: 1.2 mg/kg; range: 0.52–2.21), eight weighed 8–15 kg, (0.88 mg/kg; range: 0.54–0.99), eight weighed 16–30 kg (0.72 mg/kg; range: 0.55–0.74), and three weighed over 30 kg (0.94 mg/kg; range: 0.88–0.96). None of the animals exhibited clinical signs of adverse effects during follow-up, suggesting good tolerability at the administered doses, even in patients with systemic diseases.

**Figure 1:**
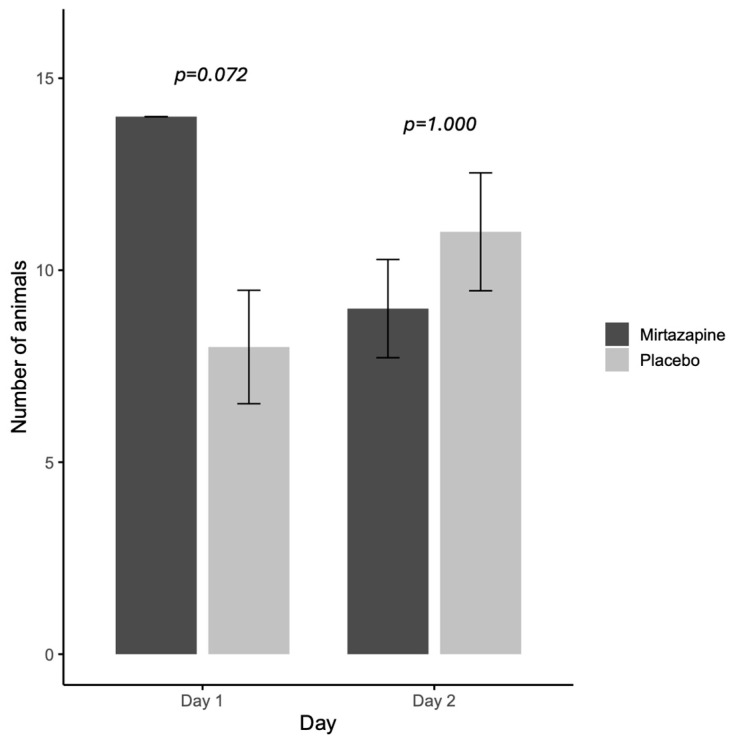
Rate of food acceptance (intake ≥ 25% of the daily portion offered) following oral administration of mirtazapine or placebo on days 1 and 2 of the study.

When evaluating the orexigenic efficacy of mirtazapine (Figure 1), on the first day all animals that received mirtazapine (*n* = 14/14; 100%) had food acceptance and this rate was higher than placebo group (7/11; 63.6%). While on the second day, there is no difference between the rate of food acceptance between groups mirtazapine (10/11) and placebo (11/14) (Figure 1).

**Figure 2:**
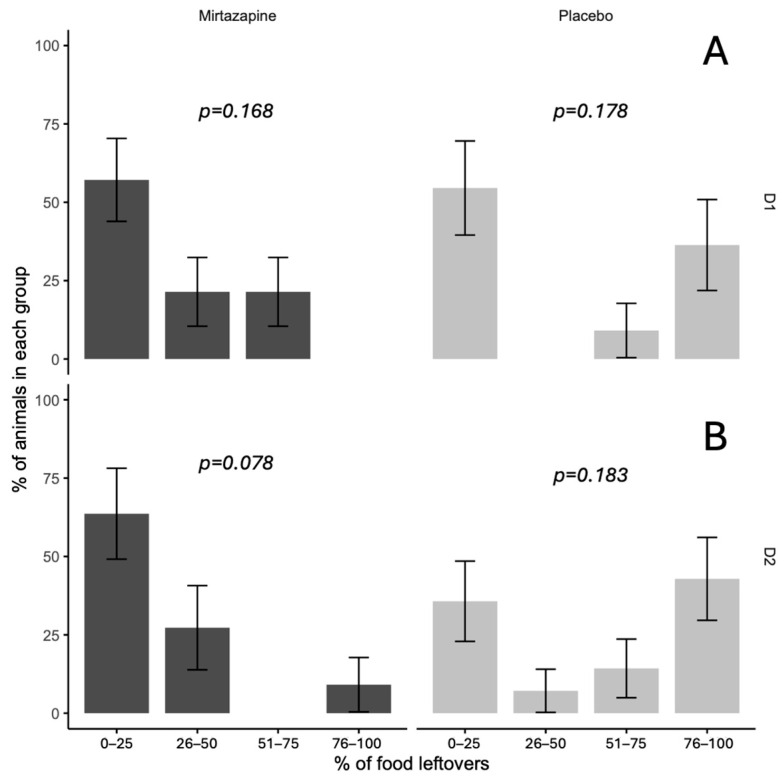
Percentage of food leftovers and distribution of animals by consumption groups following mirtazapine or placebo administration, on (**A**) first day 1 and (**B**) second day.

Analysis of leftover food revealed significant differences between treatment groups (*p* = 0.045), with mirtazapine completely preventing extreme food refusal (0% in the 76–100% category) compared to placebo (38%). In other categories, no significant differences were found (0–25%, 26–50%, 51–75%), with *p* values ranging from 0.078 to 0.183. The mirtazapine group had higher proportions in the lowest leftover food categories, while placebo exhibited greater variability, including cases of complete refusal (Figure 2).

In the mirtazapine–placebo sequence group (*n* = 14), a non-significant 5.7% reduction in food consumption was observed on Day 2 compared to Day 1 (*p* = 0.336). In the placebo–mirtazapine group (*n* = 11), there was a similarly non-significant 2.3% increase in consumption on Day 2 (*p* = 0.294) (Table 3).

Regarding the latency period, there was also no difference between days, i.e., between treatments in the same group, or between groups, i.e., on the same day (Table 4).

## 4. Discussion

Despite initial caregiver resistance to the use of feeding tubes, in the Small Animal Clinical Nutrition Service of our teaching hospital, enteral support was provided in more than 80% of clinical cases involving hyporexia, anorexia, or preemptive nutritional intervention. As noted, mirtazapine was recommended only as a secondary intervention, mainly in dogs due to few scientific evidence, either when enteral feeding was contraindicated (Study 1) or when withholding food for a brief period posed no clinical risk (Study 2), given its double-blind crossover design.

Two studies were conducted to evaluate the effects of mirtazapine on canine appetite: one retrospective and one prospective. In the retrospective study, dogs treated with mirtazapine were three times more likely to show improvement in appetite (OR = 3.06) compared to untreated dogs that voluntarily resumed food intake—an outcome consistent with previous findings in other species [18,26,27]—which had not yet been evaluated in canine patients, only in experimental studies with healthy dogs without a specific focus on appetite evaluation [28,29] or which observed an increase in appetite as a side effect in 4 from 32 dogs treated for behavioral problems [30]. Moreover, in our study dogs receiving mirtazapine exhibited greater body weight variation than those who did not respond positively to the medication, highlighting variability in individual therapeutic response.

In the prospective study, food acceptance rates were higher on the first day of treatment when dogs received mirtazapine compared to placebo, suggesting a rapid onset of orexigenic activity. However, when analyzing the effect of capsule type and treatment day individually, no statistically significant differences were observed in the percentage of food eaten between the mirtazapine–placebo and placebo–mirtazapine groups.

Because Study 1 was retrospective in nature, the mirtazapine dosages followed conservative protocols, taking into account the patients’ comorbidities. These dosages were lower than those typically recommended for healthy dogs (1.17–1.33 mg/kg q24h) [28]. Our findings showed that dogs treated with mirtazapine and those with spontaneous recovery of appetite exhibited comparable weight variation profiles, suggesting that the restoration of adequate caloric intake contributed to improved energy balance [31]. Although no statistically significant difference in weight variation was observed between mirtazapine-treated and control dogs, 68.6% of dogs receiving mirtazapine showed improved appetite, compared to 37.5% of controls. Notably, dogs in the mirtazapine-treated nonresponder subgroup experienced marked weight loss (mean = −1.5%), with even greater weight decline observed in untreated controls with persistent anorexia (mean = −2.91%). These findings underscore the detrimental impact of prolonged caloric deficit in clinically compromised patients [32].

Despite changes in food intake and body weight, no significant alterations were observed in body condition score (BCS) or muscle condition score (MCS) across the four study groups from study 1. On average, all groups maintained ideal BCS (4–5/9), but MCS remained in the moderate range. This may reflect the limited sensitivity of categorical scoring systems in detecting subtle body composition changes during hospitalization or over short timeframes [32,33].

Mirtazapine was generally well tolerated and appeared safe in the studied canine population. No adverse clinical signs related to the medication were observed during the follow-up period, even in dogs with systemic illnesses. This may be related to the fact that the pharmacokinetics of mirtazapine are different in dogs than in other species, with a shorter half-life and higher clearance rate compared to cats [28,34]. In addition, the dose of mirtazapine for cats has already been evaluated in different disease situations such as chronic kidney disease, lymphoma and hepatopathies, and not just orally. In our study, among nonresponders, most had chronic multisystemic diseases—such as renal, cardiac, or gastrointestinal disorders—that may blunt the orexigenic response. In such cases, the severity of illness may disrupt central appetite regulation pathways, limiting the efficacy of pharmacologic intervention alone [11]. For these patients, a different mirtazapine dosage could be interesting, but with this study design it was not evaluated.

The second experiment supported the short-term efficacy of mirtazapine: all dogs received mirtazapine on the first day and consumed at least 25% of their food intake. The lack of significant difference on the second day may be attributed to clinical improvement due to ongoing supportive care, or to a residual pharmacological effect, as previous studies in cats have shown that mirtazapine can remain active for up to 72 h [12]. Although a residual effect cannot be ruled out, the duration of capsule use was standardized to avoid prolonged periods of negative energy balance, as all dogs had experienced anorexia or hyporexia for 1 to 3 days prior to inclusion; in addition, the metabolization of mirtazapine seems to be faster in dogs than in cats [28].

The average latency time between mirtazapine administration and food intake was 120 min on Day 1 and 135 min on Day 2. These times are consistent with pharmacokinetic data showing peak plasma concentrations occurring 1.5–2 h after oral administration in dogs [28]. Nonetheless, orexigenic onset did not appear to differ in terms of minutes when comparing mirtazapine to placebo. When analyzing the effects of treatment day and capsule type, mirtazapine did not produce statistically significant changes in food intake, regardless of administration sequence. The high inter-individual variability observed (reflected by wide confidence intervals) suggests differences in response or the need for a larger sample size to detect subtler effects.

This is the first study to specifically evaluate the effect of mirtazapine on sick dogs with different clinical situations, with interesting results on food stimulation in these animals. However, the study has some limitations, mainly due to the fact that study 1 was retrospective; for example, 43.4% of cases were excluded due to incomplete medical records, limiting the interpretability of retrospective data. In study 2, the crossover design allowed the drug’s effect to be assessed only in the short term, preventing us from drawing conclusions regarding its potential utility in long-term management of chronic diseases, which has been demonstrated in cats but not yet in dogs. Additionally, we recognize that the timing of the first administration (Day 1 versus Day 2) may have influenced appetite response, particularly given the possible confounding effects of hospitalization and concomitant treatments received on Day 1. While the crossover model was selected to minimize interindividual variability and enhance statistical power with a limited sample size, this approach may have introduced variability linked to treatment timing. Future studies could consider alternative designs, such as parallel groups receiving standardized treatments on Day 1 (mirtazapine versus placebo), with monitoring performed entirely under hospital conditions by trained staff, to reduce confounding variables and strengthen the robustness of the findings. Taken together, these limitations suggest that while our results support the potential of mirtazapine as an appetite stimulant in dogs, prospective long-term trials under controlled conditions are necessary to confirm its efficacy and safety.

## 5. Conclusions

In conclusion, our findings reinforce that mirtazapine is a generally safe and viable appetite stimulant for dogs and can be considered a second-line intervention in cases of reduced appetite, particularly in the acute phase of nutritional recovery.

## Figures and Tables

**Table 1:** Occurrence of inappetence and management of cases attended by the Small Animal Clinical Nutrition Service between March 2016 and February 2017 and the responses about the use of mirtazapine in dogs.

Total	
Served by the Service	3063
Canine cases	2644 (83.3%)
Lack of appetite	566 (21.4%)
Enteral feeding tube	455 (80.4%)
Nasoesophageal	222 (48.8%)
Nasogastric	35 (7.7%)
Esophageal	126 (27.7%)
Mirtazapine prescription for dogs	189 (33.4%)
Excluded	82 (43.4%)
Total in the study	107 (56.6%)
Control	56 (52.3%)
Mirtazapine	51 (47.7%)

**Table 2:** Distribution of cases included in the study according to whether or not they received mirtazapine and the outcome of increased appetite.

	Mirtazapine Group *n* = 51		Control Group *n* = 56		*p*-Value
	Positive Effect	Negative Effect	Positive Effect	Negative Effect	
Appetite, *n* (%)	35 (68.6) ^a^	16 (31.4)	21 (37.5) ^b^	35 (62.5)	0.002 * ^1^
Average age, median years (range)	9.0 (4.0–13.0)	10.50 (8.5–13.0)	9.0 (2.0–11.0)	10.0 (8.0–13.0)	0.150 ^#^
Weight, kg median (range)	8.9 (5.6–19.0)	8.8 (7.8–25.68)	8.4 (5.6–23.40)	8.4 (4.7–15.7)	0.598 ^#^
BCS 9-point, median (range)	4.31 (2–9)	3.94 (1–6)	5.05 (2–9)	4.37 (1–8)	0.400 ^#^
MMS 4-point, median (range)	1.86 (1–3)	1.75 (0–3)	2.05 (0–3)	1.74 (0–3)	0.650 ^#^
Weekly weight variation, % mean (SD)	1.55 (4.82) ^a^	−1.50 (3.71) ^b^	2.62 (5.65) ^a^	−2.91 (6.75) ^c^	<0.001 ^&^
Weight maintenance, *n* (%)	6 (17.1)	4 (25.0)	4 (19.0)	10 (28.6)	0.691 *
Mirtazapine dose, median mg/kg/day (range)	0.6 (0.45–0.74) ^a^	0.6 (0.58–0.67)	0 ^b^	0	<0.001 ^#^

* Fisher’s exact test (with Freeman-Halton post hoc for 4 groups), ^1^ OR calculated for comparison Mirtazapine_Positive effect vs. Control_Positive effect–OR = 3.06, ^#^ Kruskal–Wallis test with Dunn’s post hoc ^&^ one-way ANOVA with Tukey’s post hoc, Distinct letters (^a^, ^b^, ^c^) indicate significant differences (*p* < 0.05)*,* BCS: body condition score [24]; MMS: muscle mass score [25].

**Table 3:** Percentage of food consumption on day 1 and day 2 according to each sequence group.

Group	Day 1	Day 2	*p*-Value
Mirtazapine–Placebo (*n* = 14)	69.6	63.9	0.336
Placebo–Mirtazapine (*n* = 11)	61.8	64.1	0.294
*p*-value	0.629	0.992	-

**Table 4:** Distribution of the latency period (minutes) according to the treatment sequence between mirtazapine and placebo.

Day	Day 1	Day 2	*p*-Value
Mirtazapine or Placebo (*n* = 25)	120	150	0.460
Capsule	Mirtazapine	Placebo	*p*-value
Day 1 or Day 2 (*n* = 25)	120	135	0.111

## Data Availability

The data presented in this study are available upon request from the corresponding author.

## Abbreviations

The following abbreviations are used in this manuscript:

BCS	Body Condition Score
MMS	Muscle Mass Score
OR	Odds Rations
CI	Confidence Intervals

## References

[1] Saker R.L., Remillard R.L., Hand M.S., Thatcher C.D., Remillard R.L., Roudebush P., Novotny B.J. (2010). Critical Care Nutrition and Enteral-Assisted Feeding. Small Animal Clinical Nutrition.

[2] Taylor S., Chan D.L., Villaverde C., Ryan L., Peron F., Quimby J., O’Brien C., Chalhoub S. (2022). 2022 ISFM Consensus Guidelines on Management of the Inappetent Hospitalised Cat. J. Feline Med. Surg..

[3] Taylor S. (2024). Managing the Inappetent Hospitalised Cat: International Society of Feline Medicine Guidelines. Companion Anim..

[4] Weeth L.P. (2015). Appetite Stimulants in Dogs and Cats. Nutritional Management of Hospitalized Small Animals.

[5] van den Brink W., van Bilsen J., Salic K., Hoevenaars F.P.M., Verschuren L., Kleemann R., Bouwman J., Ronnett G.V., van Ommen B., Wopereis S. (2019). Current and Future Nutritional Strategies to Modulate Inflammatory Dynamics in Metabolic Disorders. Front. Nutr..

[6] Case L.P., Daristotle L., Hayek M.G., Raasch M.F. (2011). Canine and Feline Nutrition—A Resource for Companion Animal Professionals.

[7] Johnson L.N., Freeman L.M. (2017). Recognizing, Describing, and Managing Reduced Food Intake in Dogs and Cats. J. Am. Vet. Med. Assoc..

[8] Delaney S.J. (2006). Management of Anorexia in Dogs and Cats. Vet. Clin. N. Am. Small Anim. Pract..

[9] Larsen J.A. (2023). Enteral Nutrition and Tube Feeding. Applied Veterinary Clinical Nutrition.

[10] Agnew W., Korman R. (2014). Pharmacological Appetite Stimulation: Rational Choices in the Inappetent Cat. J. Feline Med. Surg..

[11] Zollers B., Wofford J.A., Heinen E., Huebner M., Rhodes L. (2016). A Prospective, Randomized, Masked, Placebo-Controlled Clinical Study of Capromorelin in Dogs with Reduced Appetite. J. Vet. Intern. Med..

[12] Quimby J.M., Lunn K.F. (2013). Mirtazapine as an Appetite Stimulant and Anti-Emetic in Cats with Chronic Kidney Disease: A Masked Placebo-Controlled Crossover Clinical Trial. Vet. J..

[13] Quimby J.M., Brock W.T., Moses K., Bolotin D., Patricelli K. (2015). Chronic Use of Maropitant for the Management of Vomiting and Inappetence in Cats with Chronic Kidney Disease: A Blinded, Placebo-Controlled Clinical Trial. J. Feline Med. Surg..

[14] Schwasinger-Schmidt T.E., Macaluso M. (2018). Other Antidepressants. Other Antidepressants. Handbook of Experimental Pharmacology.

[15] Benson K.K., Zajic L.B., Morgan P.K., Brown S.R., Hansen R.J., Lunghofer P.J., Wittenburg L.A., Gustafson D.L., Quimby J.M. (2017). Drug Exposure and Clinical Effect of Transdermal Mirtazapine in Healthy Young Cats: A Pilot Study. J. Feline Med. Surg..

[16] Budde J.A., McCluskey D.M. (2023). Plumb’s Veterinary Drug Handbook.

[17] Ferro L., Ciccarelli S., Stanzani G., Nappi L., Angelini F., Leo C. (2022). Appetite Stimulant and Anti-Emetic Effect of Mirtazapine Transdermal Ointment in Cats Affected by Lymphoma Following Chemotherapy Administration: A Multi-Centre Retrospective Study. Animals.

[18] Poole M., Quimby J.M., Hu T., Labelle D., Buhles W. (2019). A Double-Blind, Placebo-Controlled, Randomized Study to Evaluate the Weight Gain Drug, Mirtazapine Transdermal Ointment, in Cats with Unintended Weight Loss. J. Vet. Pharmacol. Ther..

[19] Fantinati M., Trnka J., Signor A., Dumond S., Jourdan G., Verwaerde P., Priymenko N. (2020). Appetite-Stimulating Effect of Gabapentin vs Mirtazapine in Healthy Cats Post-Ovariectomy. J. Feline Med. Surg..

[20] Ozawa S., Thomson A., Petritz O. (2022). Safety and Efficacy of Oral Mirtazapine in New Zealand White Rabbits (*Oryctolagus cuniculus*). J. Exot. Pet. Med..

[21] Howard M.L., Hossaini R., Tolar C., Gaviola M.L. (2019). Efficacy and Safety of Appetite-Stimulating Medications in the Inpatient Setting. Ann. Pharmacother..

[22] National Research Council (NRC) (2006). Nutrient Requirements of Dogs and Cats.

[23] FEDIAF (2024). Nutritional Guidelines For Complete and Complementary Pet Food for Cats and Dogs.

[24] Laflamme D. (1997). Development and Validation of a Body Condition Score System for Dogs: A Clinical Tool. Feline Pract..

[25] Baldwin K., Bartges J., Buffington T., Freeman L.M., Grabow M., Legred J., Ostwald D. (2010). AAHA Nutritional Assessment Guidelines for Dogs and Cats. J. Am. Anim. Hosp. Assoc..

[26] Anttila S.A.K., Leinonen E.V.J. (2001). A Review of the Pharmacological and Clinical Profile of Mirtazapine. CNS Drug Rev..

[27] Draper J.M., Savson D.J., Lavin E.S., Feldman E.R., Singh B., Martin-Flores M., Daugherity E.K. (2022). Comparison of Effects of Capromorelin and Mirtazapine on Appetite in New Zealand White Rabbits (*Oryctolagus cuniculus*). J. Am. Assoc. Lab. Anim. Sci..

[28] Giorgi M., Yun H. (2012). Pharmacokinetics of Mirtazapine and Its Main Metabolites in Beagle Dogs: A Pilot Study. Vet. J..

[29] Yin J., Song J., Lei Y., Xu X., Chen J.D.Z. (2014). Prokinetic Effects of Mirtazapine on Gastrointestinal Transit. Am. J. Physiol.-Gastrointest. Liver Physiol..

[30] Argüelles J., Duque B., Miralles M., Bowen J., Fatjo J. (2024). Use of Mirtazapine in the Treatment of Canine Behaviour Problems: A Review of 32 Cases. Vet. Rec..

[31] Corbee R.J., Van Kerkhoven W.J.S. (2014). Nutritional Support of Dogs and Cats after Surgery or Illness. Open J. Vet. Med..

[32] Molina J., Hervera M., Manzanilla E.G., Torrente C., Villaverde C. (2018). Evaluation of the Prevalence and Risk Factors for Undernutrition in Hospitalized Dogs. Front. Vet. Sci..

[33] Leung Y.B., Cave N., Wester T.J. (2023). Loss of Body Weight and Lean Mass in Long-stay, Hospitalized Canine Patients. J. Anim. Physiol. Anim. Nutr..

[34] Richter C.M.K. (2024). The Use of Mirtazapine as an Adjunct Agent to Fluoxetine and Paroxetine in the Treatment of Canine Fear-, Anxiety-, and Aggression-Based Disorders: A Retrospective Study of 71 Cases. J. Vet. Behav..

